# In Vivo Efficacy of a Nanoconjugated Glycopeptide Antibiotic in Silkworm Larvae Infected by *Staphylococcus aureus*

**DOI:** 10.3390/insects15110886

**Published:** 2024-11-13

**Authors:** Aurora Montali, Francesca Berini, Federica Gamberoni, Ilaria Armenia, Alessio Saviane, Silvia Cappellozza, Rosalba Gornati, Giovanni Bernardini, Flavia Marinelli, Gianluca Tettamanti

**Affiliations:** 1 Department of Biotechnology and Life Sciences, University of Insubria, 21100 Varese, Italy; aurora.montali@uninsubria.it (A.M.); f.berini@uninsubria.it (F.B.); f.gamberoni@uninsubria.it (F.G.); ilaria.armenia@uninsubria.it (I.A.); rosalba.gornati@uninsubria.it (R.G.); giovanni.bernardini@uninsubria.it (G.B.); 2 Interuniversity Center for Studies on Bioinspired Agro-Environmental Technology (BAT Center), University of Napoli Federico II, Portici, 80055 Naples, Italy; 3 Council for Agricultural Research and Economics, Research Centre for Agriculture and Environment (CREA-AA), 35143 Padova, Italy; alessio.saviane@crea.gov.it (A.S.); silvia.cappellozza@crea.gov.it (S.C.)

**Keywords:** *Bombyx mori*, *Staphylococcus aureus*, nanoparticles, teicoplanin, nanoconjugated antibiotic, infection model, immune response

## Abstract

To contrast the rapid spread of antibiotic resistance in bacteria, new alternative therapeutic options are urgently needed. The use of nanoparticles as carriers for clinically relevant antibiotics represents a promising solution to potentiate their efficacy. In this study, we used *Bombyx mori* larvae for the first time as an animal model for testing a nanoconjugated glycopeptide antibiotic (teicoplanin) against *Staphylococcus aureus* infection. *B. mori* larvae might thus replace the use of mammalian models for preclinical tests, in agreement with the European Parliament Directive 2010/63/EU. The curative effect of teicoplanin (a last resort antibiotic against Gram-positive bacterial pathogens) conjugated to iron oxide nanoparticles was assessed by monitoring the survival rate of the larvae and some immunological markers (i.e., hemocyte viability, phenoloxidase system activation, and lysozyme activity). Human physiological conditions of infection were reproduced by performing the experiments at 37 °C. In this condition, nanoconjugated teicoplanin cured the bacterial infection at the same antibiotic concentration of the free counterpart, blocking the insect immune response without causing mortality of silkworm larvae. These results demonstrate the value and robustness of the silkworm as an infection model for testing the in vivo efficacy of nanoconjugated antimicrobial molecules.

## Simple Summary

Novel therapeutic treatments are urgently needed to tackle the increasing number of bacterial pathogens becoming resistant to the currently available antibiotics. One promising perspective to improve antibiotic efficacy is their conjugation to nanoparticles. Nanoconjugated antibiotics can be directed to infection sites, facilitating tissue penetration and reducing side effects. A possible bottleneck in the pipeline to develop nanoconjugated antibiotics is their in vivo testing in animal models. Since the use of mammals—i.e., the gold standard for animal testing—is limited due to economic and ethical issues, we propose a non-mammalian infection model based on the silkworm. Herein, this model is used to evaluate the efficacy of teicoplanin conjugated to iron oxide nanoparticles. Teicoplanin is a life-saving glycopeptide antibiotic for the treatment of severe infections by Gram-positive bacterial pathogens, and iron oxide nanoparticles represent a promising tool for delivering this glycopeptide antibiotic to the infection site, increasing local efficacy and reducing off-target effects.

## 1. Introduction

In the last decades, the excessive and sometimes improper use of antibiotics has favored the rapid spread of antibiotic-resistant bacteria (ARB). ARB contribute to an estimated 33,000 deaths annually in the European Union and 1.3 million deaths worldwide [1]. According to the World Health Organization (WHO), this number is expected to reach 10 million per year by 2050 [2]. Actions to counteract antimicrobial resistance (AMR) spread cannot rely exclusively on the discovery and development of new molecules with antimicrobial properties. Indeed, in recent years a limited number of chemical entities have populated the drug development pipeline, with the result that only 22 antimicrobial drugs have been approved since 2012 [3]. Innovative and improved formulations might help in potentiating the antibacterial effect of the drugs already in use and/or bypass resistance mechanisms developed by pathogenic bacteria [4]. Nanotechnologies represent a promising tool in this setting and nanomaterials carrying antibacterial molecules are today considered among the next-generation drugs [5]. In particular, iron oxide nanoparticles (IONPs) have gained attention as antibiotic carriers as they are biocompatible and non-toxic and can be moved by an external magnetic field [6,7,8,9]. Thus, loading antibiotics onto IONPs may facilitate their direct delivery to the infection site, increasing the local efficacy of the antimicrobial treatment and reducing its off-target effects [10,11]. Indeed, site-specific targeting of antibiotics nanoconjugated to IONPs might have the additional advantage of protecting human host microbiota in non-targeted body districts, reducing the risk of triggering a novel AMR response.

Development of a new antibiotic formulation, either in free or in nano-conjugated form, requires in vivo preclinical tests in animal models to evaluate its safety and efficacy before entering clinical trials [12]. Commonly, mammalian models (e.g., mice and rats) are used for this purpose. However, the restrictions imposed by the European Parliament Directive 2010/63/EU on the welfare of animals based on the 3Rs principle (i.e., “Replacement”, “Refinement”, and “Reduction”) have reinforced the need for developing alternative infection models. In this scenario, invertebrates (considered by the current scientific thinking as not being capable of experiencing suffering) may replace mammalian models for testing new formulations at different stages along the antimicrobial discovery and development pipeline [13,14]. The nematode *Caenorhabditis elegans*, the dipteran *Drosophila melanogaster,* and the lepidopterans *Galleria mellonella* and *Bombyx mori* are the invertebrates mostly used for these studies [13,15,16,17]. They offer several advantages such as the possibility of maintaining large numbers of individuals in a limited space, cheap rearing procedures, consolidated molecular tools, and gene editing systems [13]. In addition, silkworms can be reproducibly supplied by specialized centers using an artificial diet for their rearing, thus standardizing the insect stocks, and this represents a further advantage of the *B. mori* model. Consistently, the use of silkworm larvae for evaluating toxicological effects of antibiotics [18] and testing antimicrobial molecules against the major ESKAPE pathogens (i.e., *Escherichia coli*, *Pseudomonas aeruginosa*, *Staphylococcus aureus*, and *Klebsiella pneumoniae*) is well documented in the literature [19,20,21,22,23], although no previous data have been reported on nanoformulations in this insect.

To our knowledge, this is the first paper investigating the efficacy of a nanoconjugated glycopeptide antibiotic (GPA) in curing a bacterial infection in *B. mori*. The selected GPA molecule, i.e., the lifesaving teicoplanin, is part of the mainstream therapies used for the clinical treatment of relevant Gram-positive pathogens, including methicillin-resistant *S. aureus* (MRSA) and enterococci, in endocarditis, meningitis, and complicated skin and soft tissue infections [24]. Nanotechnology-inspired formulations could bypass some intrinsic limitations in GPA use, including their potential dose-dependent nephrotoxicity and poor penetration into certain body tissues and in bacterial biofilms. In this paper, teicoplanin, conjugated to IONPs through a stable amidic bond between the free amino group of the antibiotic molecule and the *N*-hydroxysulfosuccinimide ester of the bis(sulfosuccinimidyl)suberate (BS3) crosslinker [8], was administered to *B. mori* larvae systemically infected by the nosocomial Gram-positive bacterium *S. aureus.* The results indicated that the nanoconjugated teicoplanin cured the bacterial infection in vivo and that the silkworm infection model was adequate for investigating the efficacy of nanoantibiotics.

## 2. Materials and Methods

### 2.1. Materials

All chemicals and reagents reported in the following sections were purchased from Merck KgaA, Darmstadt, Germany, unless otherwise indicated.

### 2.2. Experimental Model

*B. mori* larvae (polyhybrid (126 × 57) (70 × 90)), provided by CREA-AA Sericulture Laboratory (Padova, Italy), were maintained in glass Petri dishes at 25 ± 0.5 °C, 70 ± 5% relative humidity, under a 12:12 h light:dark photoperiod. Larvae were reared with an artificial diet [25] until the end of the 4th larval instar. After the last larva-larvamolt, insects were synchronized [26] and fed with a germ- and antibiotic-free diet according to Casati et al. [27].

### 2.3. Bacterial Strain

*S. aureus* subsp. Rosenbach ATCC 6538P was grown overnight in 10 mL of Müller Hinton Broth 2 (MHB, VWR International S.r.l., Radnor, PA, USA), at 37 °C and 200 rpm. A total of 1 mL of the culture was centrifuged at 1900× *g* for 10 min at 4 °C. The cell pellet was then resuspended in an appropriate volume of sterile saline solution (0.6% *w*/*v* NaCl) to reach the desired cell concentration. The volume of saline solution to be used was calculated by measuring the optical density of the culture at 600 nm and considering that one unit of OD_600nm_ corresponded to 2.4 × 10^8^ CFU (colony forming units)/mL.

### 2.4. Synthesis of Nanoconjugated Teicoplanin

Iron oxide nanoparticles (IONPs) were synthetized using the co-precipitation method and functionalized with (3-Aminopropyl)triethoxysilane (APTES) following the protocols described in Balzaretti et al. [28] and Armenia et al. [29]. Briefly, 8.89 g of FeCl_3_ × 6H_2_O and 3.28 g FeCl_2_ × 4H_2_O were dissolved in 380 mL of water, under vigorous stirring for 30 min, while slowly adding 1.5 mL of 37% *w*/*v* HCl dropwise into the solution to completely dissolve the salts. Subsequently, 25 mL of 25% *w*/*v* NH_4_OH were added. After three washes with Milli-Q water, 40 mL of 2 M HNO_3_ were added to the particles and the solution was heated at 90 °C for 5 min. Then, the particles were separated from the reaction mixture with a magnet; subsequently, 60 mL of 0.34 M solution of Fe(NO_3_)_3_ × 9H_2_O were added. The suspension was heated at 90 °C for 30 min. Finally, the supernatant was removed and IONPs were collected with a magnet, suspended in Milli-Q water, and left in dialysis overnight against Milli-Q water. IONPs were stored at 4 °C. 

To prepare functionalized IONPs, 1.5 M solution of APTES in ethanol was added to 150 mg of IONPs and stirred for 1 h at room temperature. The temperature was then increased to 90 °C and the solution was stirred for another hour. Amino-modified IONPs (NP-APTES) were then collected with a magnet, washed several times, and suspended in Milli-Q water. Teicoplanin was conjugated to NP-APTES following the protocol described in Berini et al. [8]. Thus, 1 mL of a 4 mg/mL suspension of NP-APTES in 10 mM borate buffer pH 8.2 was added with 22.3 μg bis(sulfosuccinimidyl)suberate (BS3). The mixture was maintained under mechanical stirring for 1 h at room temperature. Subsequently, 1 mg of teicoplanin was added and the conjugation reaction was allowed to proceed for 1 h at 40 °C under mechanical agitation. The reaction was stopped by adding 500 μL of 10 mM Tris-HCl, pH 8.0. Teicoplanin-conjugated nanoparticles (NP-TEICO) were recovered using a magnet, and to determine the amount of bound teicoplanin, the supernatant was collected and analyzed at 280 nm, with a UV-Vis Jasco V-460 spectrophotometer (Jasco, Easton, MD, USA). Teicoplanin concentration in the liquid was calculated using the linear regression equation y = 0.0045x + 0.0373 (R_2_ = 0.9908). Then, the amount of antibiotic bound to NP-APTES was estimated as follows:

conjugated teicoplanin = amount of teicoplanin added to the reaction mixture − free antibiotic measured in the supernatant

NP-TEICO, resuspended in saline solution, were stored at 4 °C. Before their use, aliquots of stored material were centrifuged at 1000× *g* for 3 min, and the pellet carefully separated from the supernatant. Released teicoplanin in the liquid was measured by spectrophotometric analysis as above. The amount of GPA bound to the pelleted nanoparticles was estimated through the following equation:

conjugated teicoplanin = teicoplanin bound to nanoparticles at the time of their synthesis − teicoplanin measured in the supernatant

Additional data on NP-TEICO characterization (e.g., shape, size, size distribution, hydrodynamic diameter, polydispersity index, electrophoretic mobility, stability, and in vitro antimicrobial activity) were reported in [8].

### 2.5. Injection of Larvae

Larvae on the second day of the last larval instar were injected in the second right proleg. The injections were performed under the sterile hood by using autoclaved Hamilton 1702 LT 25 μL syringes (Hamilton, Reno, NV, USA). After the injection, the larvae were reared at 37 °C as reported in Section 2.2.

### 2.6. Determination of S. aureus Lethal Dose 50 (LD_50_) 

To determine the LD_50_, larvae were injected with 10 µL of different concentrations of *S. aureus* (corresponding to 3 × 10, 3 × 10^2^, 3 × 10^3^, 3 × 10^4^, and 3 × 10^5^ CFU), and mortality was monitored after 24 h. Uninjected larvae and larvae injected with 10 µL of sterile saline solution were used as controls. LD_50_, defined as the concentration of bacteria at which 50% of animals died within 24 h post infection, was calculated using Probit analysis [30]. Forty-five larvae in total were used for each experimental condition. Larvae were considered dead when no reaction after stimulation with a plastic tip was observed. The experiment was conducted in quintuple.

### 2.7. Effects of Nanoparticle on Larval Survival 

To evaluate the possible toxic effect of nanoparticles to *B. mori*, larvae were injected with 10 µL of 3.5, 7, 10, or 14 mg/mL NP-APTES, suspended in sterile saline solution. Control groups were represented by uninjected larvae and larvae injected with sterile saline solution. The survival rate was monitored every 24 h for three days. Thirty larvae were used for each treatment. The experiment was conducted in quadruple.

### 2.8. Administration of Teicoplanin and Nanoconjugated Teicoplanin 

The effects of free and nanoconjugated teicoplanin were evaluated by infecting the larvae with 10 µL of *S. aureus* at LD_50_ concentration and administering 10 µL of antibiotics (at a concentration equal to 8.75 µg of free or nanoconjugated teicoplanin per g of body weight, both in sterile saline solution) after 2 h, as described by Montali et al. [21]. Control groups included: uninjected larvae, larvae injected once or twice (after two hours) with 10 µL of saline solution, larvae injected with NP-APTES (10 mg/mL), and healthy larvae injected with 10 µL of free or nanoconjugated antibiotic (both at 8.75 µg/g body weight). The survival rate was checked every day for 72 h. Thirty larvae were used for each experimental condition. The experiment was performed in quadruple. 

### 2.9. Analysis of Immunological Markers

The same experimental groups described in Section 2.7 were used for analyzing the immunological markers (i.e., hemocyte viability, lysozyme activity, and phenoloxidase system activation). To elicit the activation of the immune response, larvae were injected with 3 × 10^3^ CFU of *S. aureus*, unless otherwise indicated. The hemolymph for all the experiments was collected by puncturing or cutting the second left proleg 6 or 16 h after the infection, depending on the analysis. 

#### 2.9.1. Hemocyte Viability

Hemolymph was extracted from ten surviving larvae 16 h after the first injection and diluted 1:50 with Saline Solution for Lepidoptera (210 mM sucrose, 45 mM KCl, 10 mM Tris-HCl, pH 7.0). Hemocyte viability was assessed using the CellTiter-Glo^®^ Luminescent Cell Viability Assay (Promega, Madison, WI, USA) and following the manufacturer’s instructions. Briefly, 100 µL of diluted hemolymph were added into a 96-well plate and incubated for 10 min with 100 µL CellTiter-Glo^®^ Reagent at room temperature on an orbital shaker. An infinite F200 96-well plate reader (Tecan, Männedorf, Switzerland) was used to measure luminescence. 

#### 2.9.2. Phenoloxidase (PO) System Analysis

The hemolymph collected from a pool of three surviving larvae was centrifuged at 250× *g* for 5 min at 4 °C. A total of 100 µL of supernatant was loaded into 96-well plates. The absorbance was measured by reading OD_450nm_ every 10 min for 50 min using an Infinite F200 96-well plate reader (Tecan) [31]. To evaluate the melanization rate, linear regression was performed for each ∆OD_450nm_ measurement obtained versus time [31]. The experiment was conducted in triplicate. 

#### 2.9.3. Lysozyme Activity Analysis

Lysozyme activity was analyzed as described by Montali et al. [32]. Briefly, hemolymph was extracted 6 h after infection from a pool of three surviving larvae injected with 3 × 10^3^, 3 × 10^4^, or 3 × 10^5^ CFU of *S. aureus*, transferred in an Eppendorf containing a few crystals of N-phenylthiourea to avoid melanization, and centrifuged two times at 250× *g* for 5 min and once at 1600× *g* for 10 min at 4 °C. Supernatant was collected and diluted 1:10 with sterile 30 mM phosphate buffer (38 mM KH_2_PO_4_, 61.4 mM K_2_HPO_4_, and pH 7.2). Lysozyme activity was determined by adding 100 µL of diluted hemolymph to 150 µL of 0.45 mg/mL lyophilized *Micrococcus lysodeikticus* in 30 mM phosphate buffer (OD_600nm_ of 0.6) [33]. *M. lysodeikticus* and cell-free hemolymph, both added with 30 mM phosphate buffer, were used as controls. Absorbance at 450 nm was recorded every 30 s for 10 min by using an Infinite F200 96-well plate reader (Tecan).

### 2.10. Statistical Analysis

Statistical analyses were performed using GraphPad Prism version 7.00 (GraphPad software, La Jolla, CA, USA). Data were analyzed using a One-Way ANOVA followed by a Tukey’s multiple-comparison post hoc test. Statistical differences between groups were considered significant at a *p*-value < 0.05.

## 3. Results

### 3.1. S. aureus Infection Model 

**Figure 1:**
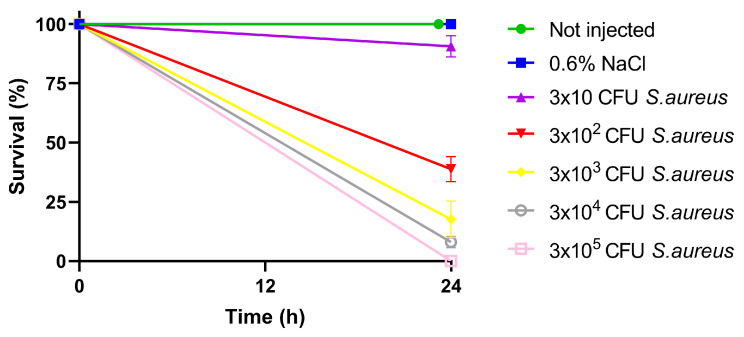
Analysis of the survival rate of *B. mori* larvae infected with different *S. aureus* bacterial cell loads (expressed as CFU) for the determination of LD_50_.

To set up the infection model, *B. mori* larvae, reared and properly synchronized as reported in Materials and Methods, were injected with increasing concentrations of *S. aureus*. The lethal bacterial dose that killed 50% of the infected larvae (LD_50_) was calculated after 24 h of incubation at 37 °C, instead of the 25 °C temperature commonly adopted for rearing larvae, to better mimic the *S. aureus* infection conditions in mammals. In these conditions, control groups (i.e., uninjected larvae and larvae injected with saline solution) showed regular development and 100% survival within a one-day time frame (Figure 1). On the contrary, in larvae infected with *S. aureus*, we observed a direct correlation between their survival rate and the injected bacterial concentration. After one day from the injection of 3 × 10 *S. aureus* CFU, 91 ± 5% of the larvae survived (Figure 1), whereas the survival rate dramatically decreased following the injection of 3x10^2^ bacterial CFU (39 ± 5%), 3 × 10^3^ CFU (17 ± 8%), and 3 × 10^4^ CFU (8 ± 2%). None of the larvae survived after 24 h from the injection of 3 × 10^5^ *S. aureus* CFU (Figure 1). LD_50_ after 24 h, calculated with Probit analysis, was shown to be 3.26 × 10^2^ CFU.

### 3.2. Efficacy of Free and Nanoconjugated Teicoplanin in Infected Larvae

Before comparing the curative effect of free and nanoconjugated teicoplanin antibiotic on infected larvae, the potential detrimental effect of nanoparticles in the silkworm larvae was investigated. Different concentrations of NP-APTES (i.e., 3.5, 7, 10, and 14 mg/mL) were injected into the larvae. The control consisted of injecting an equivalent volume of saline solution. All the groups showed 100% survival 72 h after NP-APTES administration, demonstrating that nanoparticles (in the concentration range then used for the following experiments) were not lethal for *B. mori*. 

**Figure 2:**
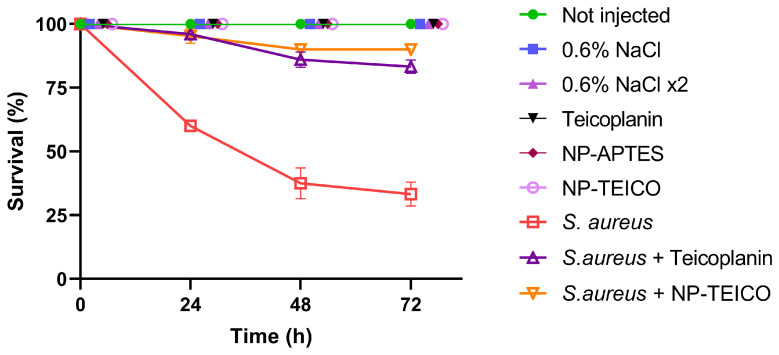
Effect of free and nanoconjugated teicoplanin (NP-TEICO) (at a concentration equal to 8.75 µg per g of body weight, both in sterile saline solution) on larvae infected by *S. aureus* at the LD_50_ bacterial concentration and on uninfected larvae as a control.

As shown in Figure 2, 100% of all of the uninfected control groups (including those injected with teicoplanin, NP-TEICO, and NP-APTES) survived in the 72 h after the injections, ruling out any detrimental effect exerted by the nanoparticles themselves and by the administered free or nanoconjugated GPA. In addition, the results reported in Figure 2 excluded any potential side effect due to the double injection procedure, validating the experimental model. Finally, the administration of either free or nanoconjugated teicoplanin (NP-TEICO) in larvae that were instead infected by *S. aureus* at the LD_50_ bacterial concentration was effective in curing the bacterial infection (Figure 2). The teicoplanin dose that was used in these experiments (8.75 µg per g of body weight) was defined in previous studies using *B. mori* to test GPAs at concentrations comparable to those used in humans to treat *S. aureus* infections [21]. Respectively, 83 ± 3% and 90 ± 2% of infected larvae survived 72 h after this treatment with free or nanoconjugated teicoplanin, in comparison to the survival rate of 33 ± 5% in untreated infected larvae. 

### 3.3. Cellular Immune Response: Hemocyte Viability

**Figure 3:**
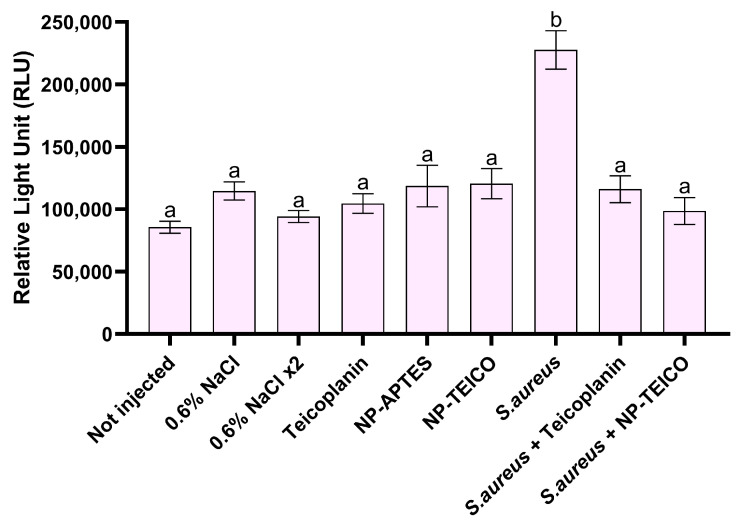
Hemocyte recruitment assayed by using a luminescence assay in larvae infected by *S. aureus* at the LD_50_ bacterial concentration and in the uninfected control groups. Values expressed as relative light unit (RLU) represent mean ± s.e.m. Different letters indicate statistically significant differences among treatments (*p* < 0.05).

Hemocytes play a crucial role in insect cellular immune response [34], and for this reason, their viability was evaluated by a luminescence assay in infected larvae treated with free or nanoconjugated teicoplanin, in parallel with the uninfected control groups. In accordance with previous studies [21,32], larvae infection by *S. aureus* led to a significant increase in the luminescence emission (Figure 3). On the contrary, free and nanoconjugated antibiotic administration blocked hemocyte activation by *S. aureus*, as confirmed by the luminescence value, which decreased to the level of the uninfected control groups (Figure 3).

### 3.4. Humoral Immune Response

#### 3.4.1. Activity of the Phenoloxidase System

**Figure 4:**
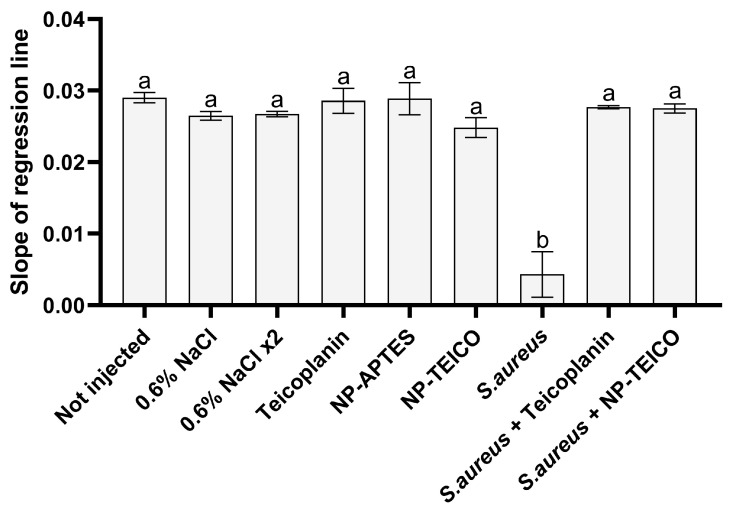
Evaluation of the PO system activity in larvae infected by *S. aureus* at the LD_50_ bacterial concentration and in the uninfected control groups. Values represent mean ± s.e.m. Different letters indicate statistically significant differences among treatments (*p* < 0.05).

In insects, the presence of pathogens in the hemolymph might activate the phenoloxidase (PO) cascade, and consequently melanin production, depending on the type and concentration of the invading microorganism [33,35]. The melanization rate in infected larvae of *B. mori* treated with free or nanoconjugated teicoplanin, in parallel with the uninfected control groups, was measured through a spectrophotometric assay. Contrary to the uninfected control groups in which no significant difference in PO activity was observed, infection of larvae with *S. aureus* led to a total impairment of the enzymatic cascade, as demonstrated by the net drop in the melanization rate. However, the treatment of insects with free or nanoconjugated teicoplanin completely restored the initial levels of PO activity (Figure 4).

#### 3.4.2. Lysozyme Activity

**Figure 5:**
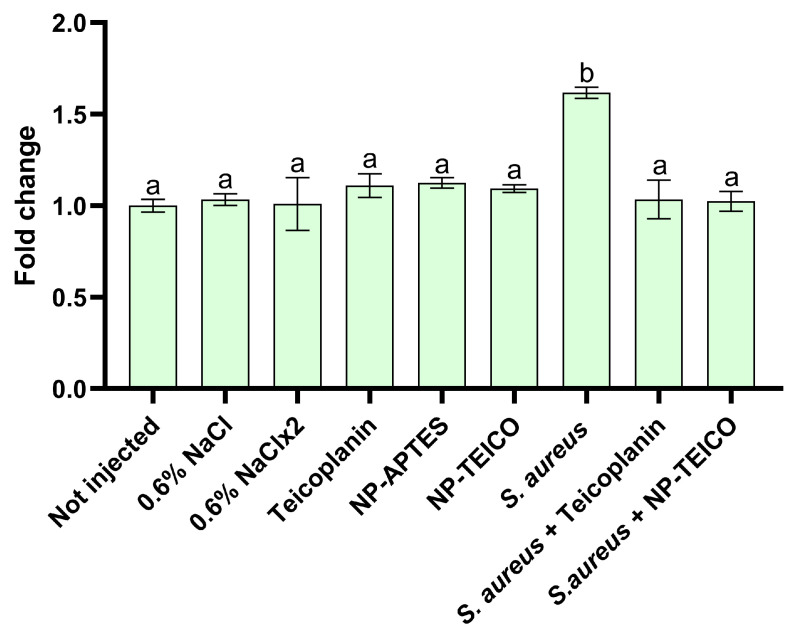
Evaluation of lysozyme activity in larvae infected by *S. aureus* (3 × 10^5^ CFU bacterial concentration) and in uninfected control groups. Values represent mean ± s.e.m. Different letters indicate statistically significant differences among treatments (*p* < 0.05).

Lysozyme is an effective mediator of the insect immune response causing bacterial pathogens’ death by hydrolysing their cell wall. Basal lysozyme activity can be measured in an insect’s hemolymph, but it is generally enhanced following an immune challenge [33,36]. Unexpectedly, the injection of larvae with 3 × 10^3^ and 3 × 10^4^ CFU of *S. aureus* suspensions did not trigger any difference in their basal lysozyme activity compared to the control groups (Figure S1). Given this result, larvae were infected with a higher bacterial load (i.e., 3 × 10^5^ CFU) to boost lysozyme activity (Figure S1 and Figure 5). In this condition, the subsequent administration of either free or nanoconjugated teicoplanin restored lysozyme activity to the basal level (Figure 5). 

## 4. Discussion

The escalating threat of antimicrobial resistance has generated an urgent need for the development of novel and effective antimicrobial strategies. Nanomaterials, when combined with antibiotics, offer a promising opportunity thanks to their unique properties, enhancing stability and efficacy and allowing the targeted delivery of the loaded antibiotic [37,38]. These attributes make nanomaterials potential game-changers in the fight against resistant bacterial infections. Iron oxide nanoparticles (IONPs) have emerged as particularly advantageous vehicles for antibiotic immobilization. The ability to direct IONPs to infection sites using an external magnetic field allows localized drug delivery, potentially reducing the required dosage and enhancing treatment efficacy through increased local drug concentration [39]. In addition, synthesis of IONPs is cost effective and scalable, and the diverse surface functionalization protocols available for these nanoparticles permit the loading of various antimicrobial agents. Remarkably, IONPs have been employed for the encapsulation or immobilization of a range of antibiotics, including teicoplanin [8,29], vancomycin [8,40,41,42], tobramycin [11], nisin [43], streptomycin, and gentamycin [44], and the potential of these antimicrobial nanoantibiotics has been confirmed by in vitro studies towards different pathogens. For instance, in our previous studies [8,29], we demonstrated that teicoplanin, once conjugated to IONPs (NP-TEICO), maintained a remarkable bactericidal activity against clinically relevant Gram-positive pathogens, such as staphylococci and enterococci, and it was active in contrasting *S. aureus* biofilm growth. 

Despite the promising in vitro activity demonstrated by nanoantibiotics, their progression to in vivo studies remans limited [45,46]. Studies in infection animal models are urgently needed for better evaluating nanoantibiotics considering factors that include toxicity, immune response, pharmacokinetics, and tissue distribution. Currently, vertebrate models, such as rodents and zebrafish, are predominantly used for this purpose [4,43]. To the best of our knowledge, there are few studies in which IONPs carrying antibiotics have been tested in infection animal models, as in the case of Chen and colleagues [47], who reported the effect of IONPs carrying vancomycin on *Clostridioides difficile* in mice infection model, demonstrating that nanoconjugated vancomycin exerted a therapeutic effect higher than free vancomycin, reducing intestinal inflammation, facilitating mucosal viability, and limiting the antibiotic side effects on the intestinal microbiota. On the other hand, biological complexity of vertebrate models, interspecies variability in response to nanoformulations, and the ethical concerns regarding animal welfare represent significant drawbacks for such in vivo studies, drastically limiting their possible extension to rapidly screening novel nanoantibiotics for clinical applications. 

In this context, the insect *B. mori* has emerged as a valuable alternative model organism for studying microbial pathogenesis and host–pathogen interactions, particularly in relation to bacterial infections [48]. Despite lacking a vertebrate-like adaptive immune system, the silkworm exhibits a highly developed innate immune response that shares significant parallels with vertebrate immune mechanisms, including Toll, IMD, and JAK/STAT pathways [49]. The conservation of these immune components and signaling pathways together with the several advantages of its use (i.e., safe handling, low rearing costs, low antibiotic amount needed, and no restrictions imposed by ethical and regulatory issues), make the silkworm a robust model for evaluating novel antimicrobial therapies with potential translational applications to vertebrate systems, including humans. The silkworm has already been reported as a reliable system to assess nanoparticle toxicity as an alternative to the cytotoxicity in vitro studies on mammalian cell lines [50,51]. Various nanoformulations of silver [52,53,54], zinc oxide [55,56,57,58], titanium oxide [59,60], magnesium oxide [61], silicon dioxide [62], and graphene oxide [63,64] have been tested in recent years in this insect. In this work, for the first time, we used *B. mori* to evaluate the toxicity of IONPs. The lack of toxicity of IONPs functionalized by APTES (NP-APTES) and of NP-TEICO injected into *B. mori* larvae herein reported, are consistent with previous findings which showed that these nanoformulations were neither toxic to different cell lines [8] nor to mice [63]. Indeed, Wang and colleagues demonstrated that the intraperitoneal injection of APTES-coated magnetic nanoparticles into ICR mice did not reduce the animal viability, maintaining the normal activity of both their liver and spleen, two of the major organs involved in the detoxification of nanomaterials in mammals [65]. 

As in the case of *B. mori*, the use of other insect models is relatively consolidated for assessing nanoparticle toxicity [54,66,67], but these invertebrates are not generally employed for evaluating the antimicrobial effect of nanomaterials. One of the few examples reported in the literature is that of *Galleria mellonella*, which was adopted to test the antimicrobial activity of colistin-carrying bimetallic silver–copper oxide nanoparticles [67] or gentamicin-loaded poly(lactide-co-glycolide) nanoparticles [68] against *P. aeruginosa* and *K. pneumoniae*, respectively. Another case is *Drosophila melanogaster*, where the antibacterial effect of ciprofloxacin-loaded chitosan nanoparticles was tested against *Salmonella* [69]. To the best of our knowledge, *B. mori* was previously used to evaluate the antimicrobial effect of two different formulations of Ag nanoparticles towards the fungus *Nosema bombycis* [70] or the bacterium *S. aureus* [71]. Consequently, this study represents the first example in which this insect was employed as an infection model to assess the efficacy of a nano-conjugated antibiotic. 

Our results demonstrated that nanoconjugated teicoplanin cured *S. aureus* infection in *B. mori* larvae with the same therapeutic effect as the equivalent dose of free teicoplanin. Taking into consideration that different parameters, such as the cellular uptake and internalization, are critical for developing a novel nano-antibiotic, our investigation was not limited to the evaluation of larvae survival, but it also covered a series of immunological markers. In fact, if the immune system cells (i.e., hemocytes) are stimulated, the efficacy of the antibacterial treatment could be reduced, and a higher dose of the nanoconjugated antibiotic would be needed. In vertebrates, macrophages play a central role in responding to nanoparticle injections due to their potent phagocytic activity and ability to efficiently internalize and clear foreign particles [72,73]. Previous in vitro studies involving macrophages exposed to various concentrations of IONPs or NP-TEICO demonstrated a dose-dependent uptake, leading to significant cytoplasmic engulfment, without any apparent effect on cell viability [8]. As mammalian macrophages, hemocytes in *B. mori* perform functions such as phagocytosis, nodulation, and encapsulation [34]. Notably, no proliferation of these cells was observed following the injection of the nanoparticles, whether conjugated with the antibiotic or not, nor of the free teicoplanin. This result was mirrored in the larvae infected with *S. aureus* treated with the nanoantibiotic, suggesting the effectiveness of NP-TEICO in contrasting the infection in *B. mori* larvae. Additionally, the humoral response, monitored by the prophenoloxidase system activation and the lysozyme activity, confirmed that the larvae’s immune system was not significantly stimulated by the injected nanosystem, irrespective of the presence of bacterial infection. The minimal or absent activation of the larval immune system indicates that the nanoconjugated teicoplanin herein employed exhibits its antibacterial activity (comparable to the free antibiotic) not only in vitro but also in vivo, paving the way for its further investigation for clinical applications.

## 5. Conclusions

The results obtained in this work demonstrated that silkworm infection model could help in predicting in vivo efficacy and toxicity of nanoconjugated antibiotics, limiting the number of mammals to be used in preclinical studies and consequently reducing the ethical and economic issues of animal testing. In vivo studies in larvae infected by *S. aureus* confirm that the nanoconjugated teicoplanin retains the same antimicrobial activity of the free antibiotic, as predicted by in vitro antimicrobial tests, without causing any toxicity effect to the animals. 

## Data Availability

The datasets generated for this study are available upon reasonable request to the corresponding author.

## Supplementary Materials

The following supporting information can be downloaded at: https://www.mdpi.com/article/10.3390/insects15110886/s1, Figure S1: Evaluation of the lysozyme activity in larvae infected with different *S. aureus* concentrations and in uninfected larvae as a control.
