# Supplementary material for: In Vivo Efficacy of a Nanoconjugated Glycopeptide Antibiotic in Silkworm Larvae Infected by Staphylococcus aureus
Source: Insects. 2024 Nov 13;15(11):886. doi: 10.3390/insects15110886 (PMC11595181; doi:10.3390/insects15110886)

Figure S1: Evaluation of the lysozyme activity in larvae infected with different *S. aureus* concentration and in uninfected larvae as a control. Values represent mean ± s.e.m. Different letters indicate statistically significant differences among treatments ($p < 0.05$).

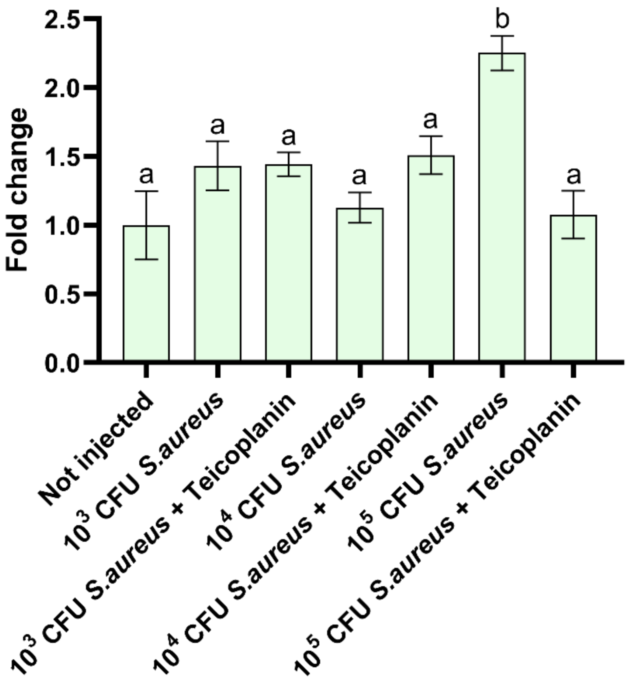

Supplement: Supplementary file 1 [file insects-15-00886-s001.zip › insects-3274306-supplementary.pdf]
